# ATRX Dysfunction Induces Replication Defects in Primary Mouse Cells

**DOI:** 10.1371/journal.pone.0092915

**Published:** 2014-03-20

**Authors:** David Clynes, Clare Jelinska, Barbara Xella, Helena Ayyub, Stephen Taylor, Matthew Mitson, Csanád Z. Bachrati, Douglas R. Higgs, Richard J. Gibbons

**Affiliations:** 1 MRC Molecular Haematology Unit, Weatherall Institute of Molecular Medicine, University of Oxford, John Radcliffe Hospital, Oxford, United Kingdom; 2 Computational Biology Research Group, Weatherall Institute of Molecular Medicine, University of Oxford, John Radcliffe Hospital, Oxford, United Kingdom; 3 School of Life Sciences, University of Lincoln, Brayford Pool, Lincoln, United Kingdom; Bellvitge Biomedical Research Institute (IDIBELL), Spain

## Abstract

The chromatin remodeling protein ATRX, which targets tandem repetitive DNA, has been shown to be required for expression of the alpha globin genes, for proliferation of a variety of cellular progenitors, for chromosome congression and for the maintenance of telomeres. Mutations in ATRX have recently been identified in tumours which maintain their telomeres by a telomerase independent pathway involving homologous recombination thought to be triggered by DNA damage. It is as yet unknown whether there is a central underlying mechanism associated with ATRX dysfunction which can explain the numerous cellular phenomena observed. There is, however, growing evidence for its role in the replication of various repetitive DNA templates which are thought to have a propensity to form secondary structures. Using a mouse knockout model we demonstrate that ATRX plays a direct role in facilitating DNA replication. Ablation of ATRX alone, although leading to a DNA damage response at telomeres, is not sufficient to trigger the alternative lengthening of telomere pathway in mouse embryonic stem cells.

## Introduction

ATRX is an SNF2-related chromatin remodelling protein which acts with the histone chaperone DAXX to insert the variant histone H3.3 into telomeric and pericentromeric heterochromatin [1], [2]. Constitutional mutations in ATRX give rise to alpha thalassaemia mental retardation, X-linked (ATR-X) syndrome [3]. The affected males exhibit severe intellectual disability, and multiple congenital abnormalities involving genital and skeletal development, as well as a characteristic facial appearance and many have alpha thalassaemia an anaemia which, in these individuals, is due to reduced alpha globin gene expression. Extensive studies in mouse have shown that absence of full- length ATRX leads to defective development of the trophoblast [4], loss of neurons in the CNS [5], lack of proliferation of myoblasts [6] and Sertoli cells [7] as well as abnormal mitosis [8] and meiosis [9]. It is unclear however how all these diverse effects result from ATRX dysfunction.

An important recent finding is that ATRX localises to G-rich tandem repeats including interstitial repeats and telomeres during S phase [10]. These sequences are thought to form secondary structures such as G quadruplex (G4) and indeed ATRX binds G4 *in vitro*. In the absence of ATRX there is an increase in DNA damage at telomeres [11] raising the possibility that ATRX is required for the replication of these unusual DNA templates and that ATRX deficiency leads to replicative stress.

Here we confirm and extend these important observations in a primary mouse cell line knocked out for ATRX and demonstrate a defect in S phase progression following an aphidicolin block, sensitivity to hydroxyurea, increased stalling of replication and an increase in double strand breaks (DSBs) as detected by 53BP1 foci and a neutral comet assay. These aspects of ATRX function may be particularly important at telomeres. It has previously been shown that, in telomerase negative tumour cells, viability is maintained via an alternative pathway (ALT) to lengthen telomeres. Furthermore, tumours with the ALT phenotype frequently harbour acquired somatic mutations in the ATRX gene [12], [13], [14]. Importantly, here we show that disruption of ATRX alone, in mouse embryonic stem cells, is not sufficient to trigger ALT and consequently other factors must contribute to this pathway. Here we used co-immunoprecipitation and co-localisation, to identify other factors that interact with ATRX and found that in wild-type cells, endogenous ATRX associates with the MRN complex which is also known to play a part in generating the ALT phenotype. This study has further developed the evidence showing that ATRX plays a direct role in DNA repair, and together with MRN, may play an important role in the ALT pathway.

## Results

### Knockout of ATRX induces a DNA damage response in mouse embryonic stem cells

**Figure 1:**
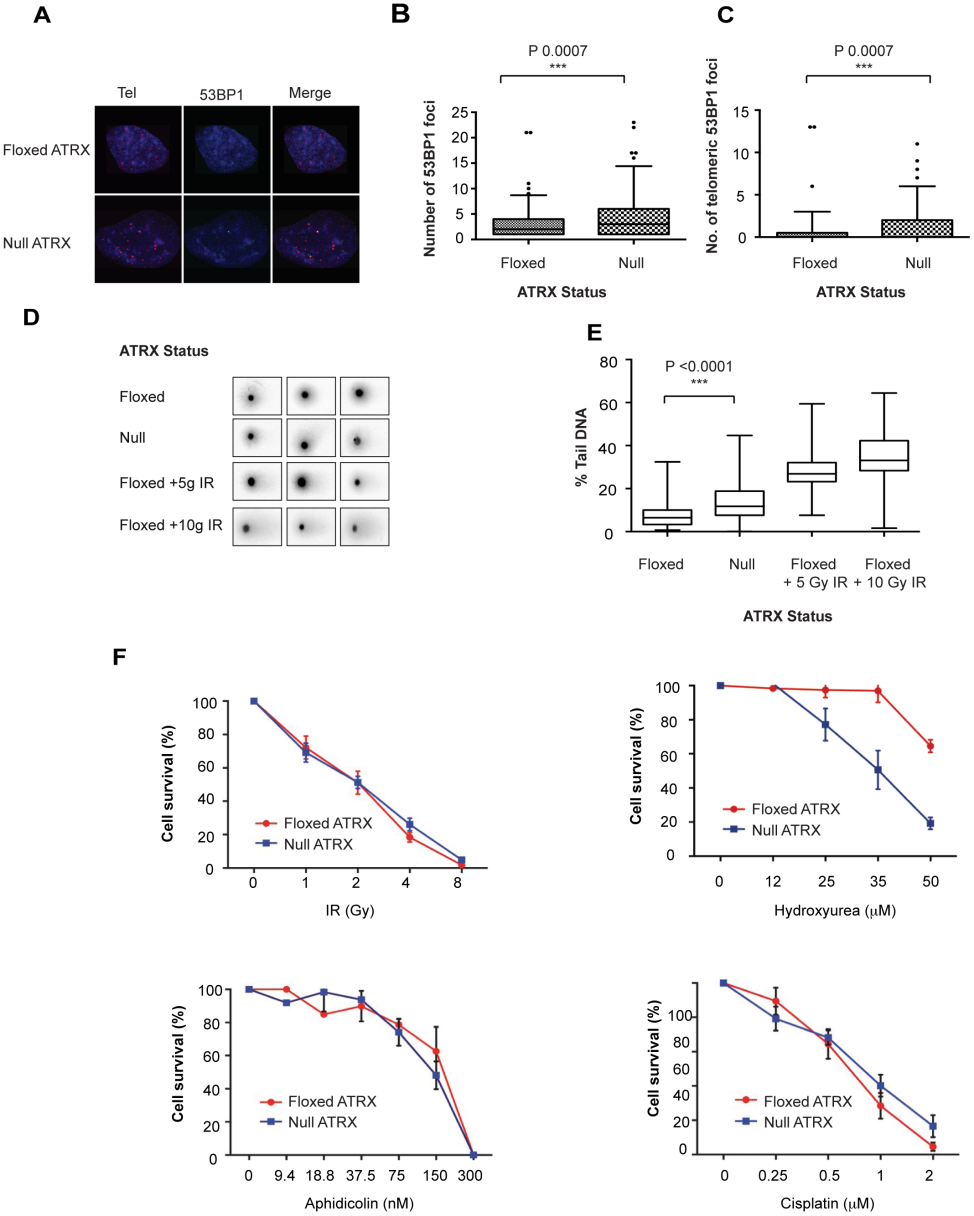
Loss of ATRX triggers a DNA damage response. (A) Representative images for immuno-FISH analysis showing colocalisation between 53BP1 and telomeres. Full data sets are available at http://sara.molbiol.ox.ac.uk/public/staylor/53BP1_Tel2 and http://sara.molbiol.ox.ac.uk/public/staylor/53BP1_Tel3. (B),(C) 53BP1 foci and 53BP1 telomere colocalising foci were scored using the JACoP plugin for ImageJ from a total of 105 *Atrx^flox^* and 115 *Atrx^null^* cells. Statistical significance was determined using a Mann Whitney test. (D) Representative images of COMETs. (E) Quantitation of DNA DSBs by COMET assay represented by the proportion of DNA in the COMET “tail” (n = 50). Exposure to gamma-irradiation was used as a positive control. (F) Cellular sensitivity of *Atrx^flox^* and *Atrx^null^* mES cells to ionising radiation (IR), hydroxyurea (HU), Aphidicolin and Cisplatin as measured by clonogenic survival assay. Error bars indicate ± SEM from a minimum of three independent experiments.

We have previously generated mouse embryonic stem (mES) cell lines conditionally deleted for full length ATRX (*Atrx^null^*) with their wildtype counterparts (*Atrx* ^flox^) mES cells [4]. Using these cell lines we sought to further characterise the cellular function of ATRX. In agreement with previous reports, loss of ATRX was associated with an increased DNA damage response, as detected by an increase in 53BP1 foci as well as an increase in telomeric 53BP1 foci (Figure 1A, B and C). We confirmed a higher frequency of DSBs, specifically, in the ATRX knockout using a neutral comet assay (Figure 1D and E). To investigate the origin of the increased DSBs in the ATRX knockout cells, clonogenic sensitivity assays were performed with a range of DNA damaging agents and DNA replication inhibitors. There was a modest increase in sensitivity in the *Atrx* ^null^ cells upon treatment with the DNA replication stress inducing drug hydroxyurea (HU) but no response to other DNA replication inhibitors (camptothecin and aphidicolin) or DNA damaging agents (gamma-irradiation (IR) and cisplatin) (Figure 1F). Whilst camptothecin inhibits topoisomerase I and aphidicolin inhibits DNA polymerase, HU inhibits DNA replication by depleting dNTPs, resulting in extended regions of single-stranded DNA [15], likely leading to an increased probability of forming G4 structures. The specific sensitivity of the *Atrx* ^null^ cells to HU is therefore consistent with a role for ATRX in processing G4 structures, as previously hypothesised [16].

### ATRX is required for proficient S phase progression by limiting fork stalling

**Figure 2:**
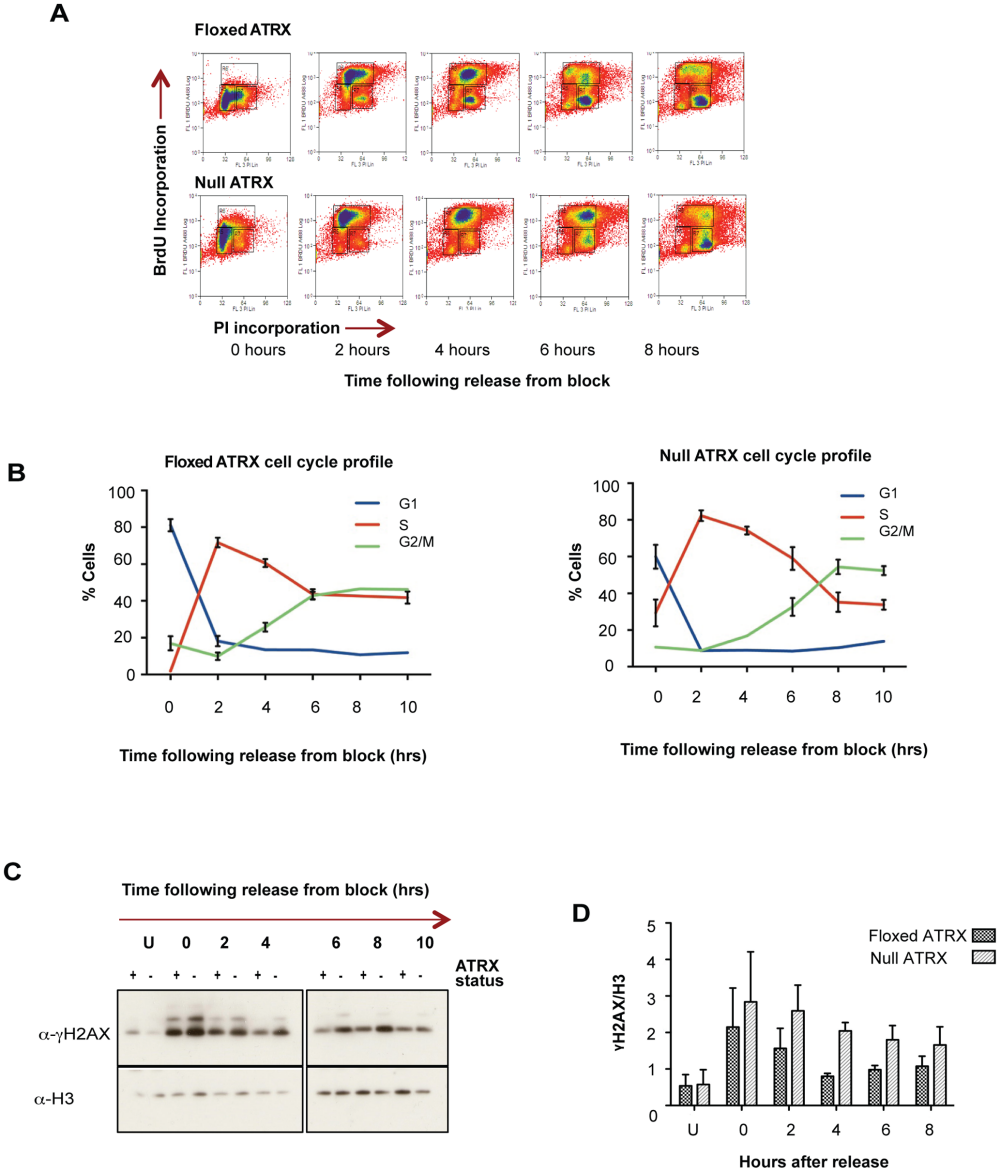
Loss of ATRX results in a prolonged S-phase. (A) Cell cycle profile for *Atrx^flox^* and *Atrx^null^* mES cells. (B) Percentage of cells in G1, S and G2/M cells at various time points following release from G1 block are shown as determined by FACs analysis. Error bars indicate ± SEM from three independent experiments. (C) Western blot and quantitation from 3 biological replicates (D) to assess levels of gamma-H2AX in histones purified from *Atrx^flox^* and *Atrx^null^* mES cells at the indicated time points. This showed an elevated DDR, reflected by elevated gamma-H2AX, in *Atrx^null^* cells as compared to the *Atrx^flox^* cells. U  =  unsynchronised cells. Histone H3 is shown as the loading control. Error bars indicate ± SEM.

The existence of a DNA replication defect upon loss of ATRX was confirmed in a number of ways. *Atrx* ^flox^ and *Atrx* ^null^ mES cells were blocked at the G1/S boundary with aphidicolin, released, and the progression through S-phase was monitored by fluorescence activated cell sorting (FACS) showing that S-phase was markedly prolonged in the *Atrx* ^null^ cells (Figure 2A and B). Interestingly, 6–10 hours post block an increased number of cells (∼20%) staining for BrdU in-between the S and G2 populations in the *Atrx* ^null^ cells became evident, most likely indicating a defect in late S-phase (Figure 2A, Figure S1A and S1B). Moreover, Western blots prepared from histones extracted in parallel and blotted for gamma-H2AX demonstrated that the prolonged S-phase in *Atrx* ^null^ cells was correlated with an elevated DDR in comparison to the *Atrx* ^flox^ cells, despite a similar initial DDR after aphidicolin treatment (Figure 2C and D). Comparable results were obtained with independently isolated *Atrx^flox^* and *Atrx^null^* clones, excluding the possibility that these differences were attributable to clonal variations (Figure S1C and S1D). We have previously reported that unchallenged, asynchronous *Atrx* ^null^ mES cells do not show alterations in cell cycle profile relative to wild type cells [4], suggesting that an aphidicolin block potentiates a more severe replication defect upon loss of ATRX.

**Figure 3:**
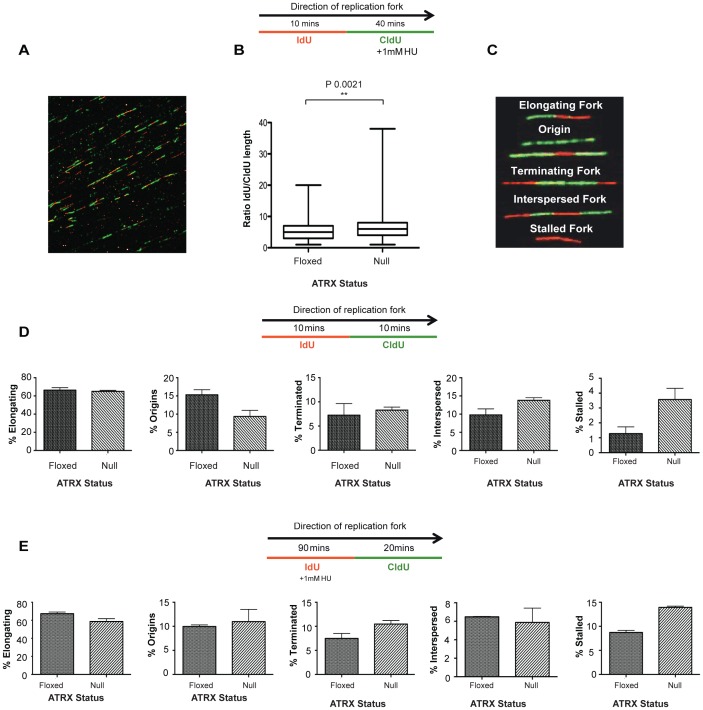
ATRX deficient cells show an increase in replication fork stalling and DNA double strand breaks. (A) Representative image of actual fibres from *Atrx^flox^* mES cells. (B) Replication fork processivity in *Atrx^flox^* and *Atrx^null^* mES cells shown as a box whisker plot of the ratio in length of IdU and CldU labelled DNA for individual replicons (n = 259 for Flox and 359 for Null). Statistical significance was determined using a Mann Whitney test. (C) Representative images of five classes of replication intermediates identifed by DNA fibre analysis in this study. (D), (E) Relative frequency of replication intermediates in *Atrx^flox^* and *Atrx^null^* mES cells without (D) and with hydroxyurea treatment (E) during the IdU pulse. Over 1000 fibres totalled from three independent replicates were scored per experiment and error bars indicate ± SEM.

Replication fork progression was next analysed at single molecule resolution using DNA fibre analysis, a technique in which replicating cells are sequentially labelled with nucleotide analogues (iododeoxyuridine (IdU) and chlorodeoxyuridine (CldU)), lysed, the DNA fibres spread on glass slides, probed with specific antibodies and visualised by fluorescence microscopy [17] (Figure 3A). We initially assessed the effect of HU treatment on replication fork processivity in *Atrx* ^flox^ and *Atrx* ^null^ cells using this technique. Cells were pulsed for 10 minutes with IdU, followed by a 40-minute incubation with CldU and 1mM HU. For each replication fork the processivity was determined as the ratio of the length of IdU:CldU labelled DNA; higher ratios corresponding to lower processivity. While the majority of replication forks exhibited a comparable reduction in processivity upon HU treatment (Figure 3B), the distribution was skewed with a significantly higher (p = 0.0021) frequency of very slow replicating forks in the *Atrx* ^null^ cells. This indicates that, although not required globally for replication fork processivity, efficient replication of a subset of genomic loci is dependent on ATRX. In addition, this DNA fibre technique allows for the identification five major replication intermediates; elongating forks, origin firing, termination events, interspersed origins and stalled replication forks (Figure 3C) [17], [18], [19]. A reproducible increase in the proportion of stalled replication forks was observed in the *Atrx* ^null^ cells relative to the *Atrx* ^flox^ cells (Figure 3D). After replication stress was induced by treatment with HU during the IdU pulse a marked increase in the frequency of stalled replication forks was observed in both the *Atrx* ^flox^ and *Atrx* ^null^ cells (Figure 3E). A small decrease in origin firing was observed in the *Atrx* ^null^ cells (Figure 3D) but this was not apparent upon treatment with HU in the IdU pulse (Figure 3E). No consistent change was observed in the occurrence of the other replication intermediates. Comparable results were obtained using the second *Atrx* ^flox^ and *Atrx* ^null^ clones (Figure S1E), suggesting that the differences were not attributable to a clonal effect. It is apparent that in the absence of ATRX, DNA replication is perturbed with an extended S-phase, an increase in stalled forks and a DDR, reflecting the presence of DSBs presumably arising from collapse of the replication fork.

### Loss of ATRX function does not affect telomere maintenance in mouse ES cells

**Figure 4:**
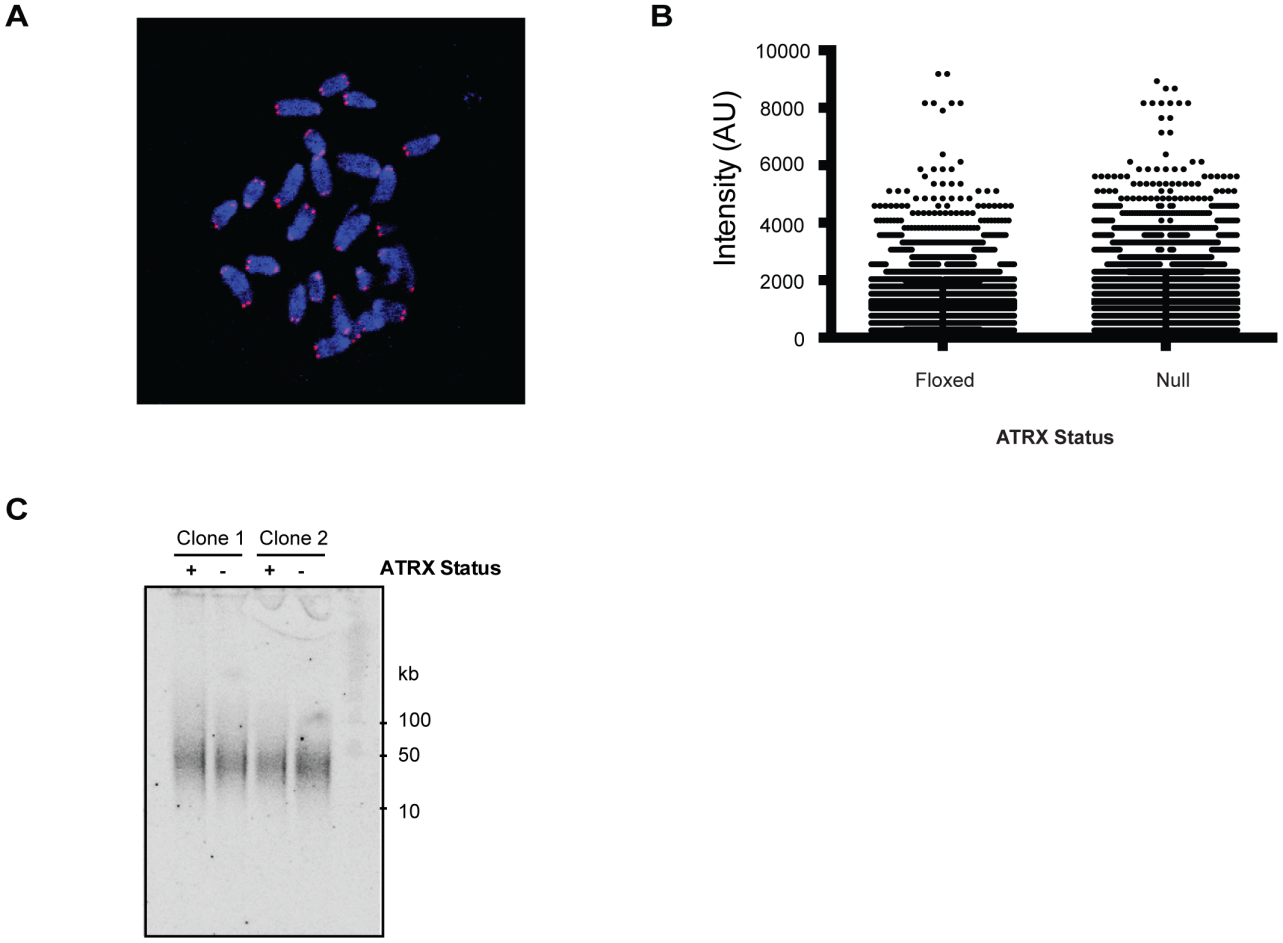
Loss of ATRX does not affect telomere maintenance in mouse ES cells. (A) Representative image of a telomere FISH performed on a metaphase spread. (B) Quantitation of telomere fluorescence intensity in Atrx*^flox^* and Atrx*^null^* mES cells from >7000 telomeres for each cell type. (C) Terminal restriction length analysis of two independent clones (1, IF12 and 2, IG11) after digestion with HinfI and RsaI. Digested DNA was probed with a radiolabelled telomeric repeat.

It has recently been shown that ATRX mutations or the absence of ATRX protein is an almost invariant finding in cell lines exhibiting the ALT phenotype and that various cancers with features consistent with the ALT phenotype are frequently associated with mutations in ATRX, DAXX and/or H3.3 [12], [14], [20]. Since replication fork stalling is a known trigger of recombination, the possibility that ATRX may normally repress this pathway in primary mouse ES cells was next considered. No marked changes in telomere length were detectable in *Atrx* ^null^ cells relative to the *Atrx* ^flox^ cells using both quantitative FISH, which gave median telomere intensities of 1020 (A.U.) for both *Atrx* ^flox^ and *Atrx* ^null^ cells (Figure 4A and B) and terminal restriction fragment length (Figure 4C) analyses. ALT is characteristically associated with heterogeneous telomere lengths and these data therefore infer that knockout of ATRX in mouse ES cells is insufficient to trigger the ALT pathway. The cellular context, including the telomerase negative status of cells, must therefore be an important additional factor in the development of the ALT pathway. Consistent with this, knockdown of ATRX in HeLa cells also fails to initiate the ALT pathway [20].

### ATRX interacts with the MRN complex

**Figure 5:**
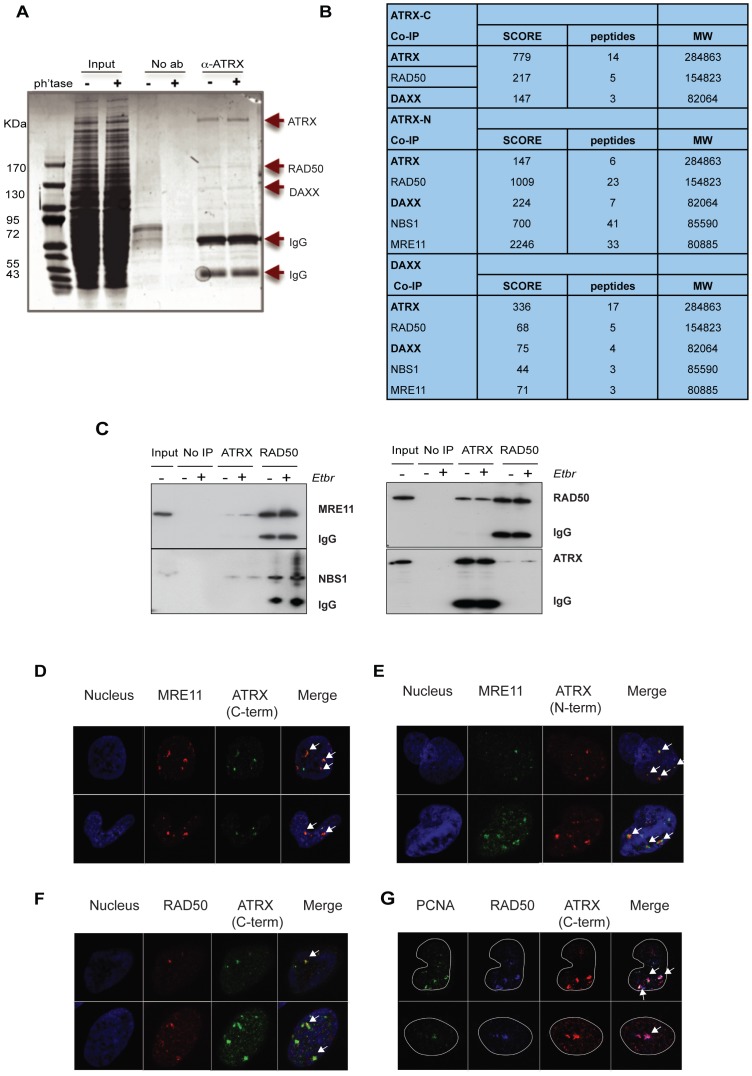
ATRX interacts with the MRN complex in replicating cells. (A) Coomassie blue stained gel showing proteins immunoisolated from HeLa nuclear extract using anti-ATRX (H300) antibody. ‘No ab’ was performed using protein A beads only. HeLa nuclear extract pre-treated with λ phosphatase was used to assess phosphorylation dependence of the interaction. (B) Table showing identification of the three major proteins immunoisolated by mass spectrometry in the different IPs. Peptides were matched on the Mascot search engine for each of the proteins. (C) Immunoblots confirming presence of RAD50, ATRX, MRE11 and NBS1 in the proteins immunisolated by the ATRX antibody from HeLa nuclear extract. As a positive control a second reverse immunoprecipitation was performed using a RAD50 (H300) polyclonal antibody. ‘No IP’ was performed using protein A beads only. (D), (E) Representative images for immunofluorescence showing co-localisation between ATRX and MRE11 using C-terminal specific (D) and N-terminal specific (E) ATRX antibodies. (F) Representative images for immunofluorescence showing co-localisation between RAD50 and ATRX and 3 way co-localisation with PCNA (G). Full data set for the co-localisation of ATRX/RAD50 in HeLa cells is available at http://sara.molbiol.ox.ac.uk/public/staylor/ATRX_MRN_PCNA/.

It is therefore clear that loss of ATRX function alone is insufficient to trigger the ALT pathway. To explore further the mechanism by which ATRX prevents replication stress/genomic instability and suppresses the ALT pathway we used co-immunoprecipitation to identify proteins interacting with endogenous ATRX. Using a polyclonal antibody raised to the C-terminus of ATRX (H300), ATRX interaction partners were immune-isolated from a HeLa nuclear extract and resolved by SDS PAGE gel electrophoresis. Three major bands were analysed by mass spectrometry (Figure 5A). In addition to ATRX and its known partner DAXX, a third protein (RAD50) was identified as a novel interaction partner. The co-immunoprecipitation reaction was repeated using an antibody raised to the N-terminus of ATRX (39F) and in this case all immune-isolated protein was subject to identification by mass spectrometry. RAD50, MRE11 and NBS1, the three components of the MRN complex [21], were identified (Figure 5B). Moreover, immunoprecipitation with an antibody to DAXX also recovered RAD50, MRE11 and NBS1 (Figure 5B).

Immunoblotting with RAD50, MRE11 and NBS1 specific antibodies confirmed the identification of these proteins as ATRX interaction partners (Figure 5C). These interactions were maintained in the presence of ethidium bromide, suggesting that the interactions were direct rather than DNA mediated (Figure 5C). The interaction between ATRX and RAD50 was confirmed in the reverse co-immunoprecipitation reaction using a RAD50 monoclonal antibody (Figure 5C).

The MRN complex has many known functions including: double strand break repair (via both HR and NHEJ) and the restart of stalled replication forks [22], [23]. We addressed whether ATRX normally co-localises with the MRN complex by dual indirect immuno-fluoresence in HeLa cells. Approximately 5 – 10% of unsynchronised HeLa cells exhibit nuclear foci containing both RAD50 and MRE11 associated with DNA damage. In 88% of these cells, ATRX co-localised with at least one of these RAD50/MRE11 foci (Figure 5D, E and F). Furthermore, all subnuclear foci in which ATRX was associated with RAD50/MRE11 were found in nuclei which stained positive for PCNA (Figure 5G), indicating that the association of ATRX and the MRN complex occurs during S phase. Analogous results were obtained in mES cells, suggesting that this interaction is not confined to HeLa cells (Figure S2). Together these data provide evidence that the ATRX/DAXX complex interacts with the MRN complex during DNA replication and is likely to play a direct role in facilitating this process.

## Discussion

Here we show that a conditional deletion of full length ATRX in primary mouse ES cells undergoing replicative stress, leads to a prolongation in S-phase, concomitant accumulation of gamma-H2AX and an increase in fork stalling. Notably, loss of ATRX elicits an apparent defect in late S phase, which likely includes replication of repetitive, heterochromatic sites, known targets of ATRX binding [10]. In accordance with this, ATRX is not generally required for fork processivity but instead appears to be required for the efficient replication of a subset of genomic loci. Although in this study we were unable to identify these sites it is likely that they represent late replicating ATRX target sites.

This model has gained support from a number of complementary studies. Ablation of ATRX in mouse myoblasts delayed their progression in S phase and led to an accumulation of DNA damage including telomere fragility [6] and similar findings were observed in a study involving neuroprogenitors [24]. In a further advance, a somatic knockout of ATRX in a colorectal cell line exhibited increased sensitivity to hydroxyurea and aphidicolin suggesting that in the absence of ATRX there is an increase in replicative stress in this cellular context [25]. Taken together with the findings presented here it is clear that ATRX has a general role in facilitating DNA replication in multiple cellular environments and that this may in part account for why loss of ATRX function results in such disparate pathologies. Given the role reported here in ES cells it is likely that this function is also relevant in normal development.

A large proportion of ATRX target sites are predicted to adopt non-B form secondary structures, including the G-quadruplex conformation [10]. Accumulating evidence suggests that the presence of such secondary structures serve as a barrier to replication fork progression, leading to fork stalling or collapse [26], [27], [28]. It is therefore tempting to think that ATRX is responsible for preventing the formation and/or accumulation of such structures at its repetitive target sites, thereby facilitating replication. This hypothesis is supported by the particular sensitivity of *Atrx^null^* cells to hydroxyurea, a replication inhibitor that increases the likelihood of G4 formation. Moreover, in line with these observations, *Atrx^null^* neuroprogenitor cells have recently been shown to be sensitive to the G-quadruplex stabilising ligand telomestatin [24], lending weight to the notion that ATRX aids the replication of G-quadruplex structures. Here we have shown that ATRX does not itself appear to possess G-quadruplex unwinding activity (Figure S3) suggesting that ATRX must overcome these impediments indirectly, perhaps by facilitating histone H3.3 deposition to maintain DNA in the B-form, or alternatively by promoting fork bypass via a process such as template switching. Further work is needed to determine where ATRX functions, physically and temporally, in relation to the replication fork.

Interestingly, a role for ATRX in facilitating replication through potential G-quadruplex forming sequences may shed light on its recently ascribed role as a tumor suppressor in a specific subset of malignancies that depend on a telomerase-independent pathway of telomere maintenance called the ‘alternative lengthening of telomeres’ (ALT) pathway [12], [14], [20]. ALT is thought to depend on recombination between telomeric sequences [29] and therefore one could envisage that the presence of G-quadruplex structures in the absence of ATRX and subsequent fork stalling may serve as a trigger for HR. We note that telomere length remains largely unperturbed in the *Atrx^null^* cells, thereby suggesting that loss of ATRX alone is not sufficient to trigger ALT in this cellular context. It is therefore obviously of interest to determine the additional requirements for ALT activation.

By co-immunoprecipitation we demonstrate an interaction between endogenous ATRX and components of the MRE11-RAD50-NBS1 (MRN) complex during S phase and a similar finding, using an expressed tagged ATRX transgene, has recently been reported[25]. Like ATRX, the MRN complex localises to telomeres during the S and G2 phases of the cell cycle [30], [31] and interestingly G-quadruplexes are a preferred substrate for MRE11[32]. MRN has many known functions important for genomic stability and replication, including the repair of double strand breaks (via both HR and NHEJ) and the restart of stalled replication forks. Furthermore the MRN complex is required for ALT [33], [34] It is likely that the interaction between ATRX and MRN is important to the role of ATRX in facilitating replication and genomic stability [22], [23]. It will be of considerable interest in future studies to determine the mechanism by which ATRX suppresses ALT and whether the interaction between ATRX and MRN plays a role in this process.

## Materials and Methods

### Cell cycle synchronisation and analysis

Cell cycle synchronisation was performed using a thymidine aphidicolin double block as previously described [35]. 30 mins prior to each time point cells were incubated with 10 μM BrdU. BrdU incorporation and DNA content were assessed by FACs analysis using propidium iodide staining, anti-BrdU antibody (Abcam ab6326) and goat anti-rat A488 secondary antibody (Invitrogen A11006). Histones from each time point were solubilised in 2M HCl and precipitated in acetone overnight at −20°C. Western blotting was performed using anti-gamma-H2AX (Millipore 05-636) and anti-histone H3 antibody (Abcam ab1791).

### COMET assay

mES cells were treated with ionising radiation at the dosages specified and the Comet assay was performed as described in [36] with the following modifications. Following electrophoresis and washes in Tris buffer slides were washed in 70% ethanol and air-dried prior to staining with SybrGold (Invitrogen S1-494). 50 COMETs from each cell type/dosage were analysed using Komet 6 (Andor) software.

### Immunoisolation of ATRX interaction partners

Immunoprecipitations were performed from 2 to 4 mg HeLa nuclear extract (Cilbiotech) in IP buffer (20 mM HEPES pH 7.4, 1% Triton X-100, 150 mM NaCl, 1 mM EDTA, 1 mM EGTA + protease inhibitor cocktail). Immunoprecipitations were performed overnight at 4°C using 3 μg anti-ATRX antibody (sc-15408) and 4 μg anti-RAD50 antibody (Abcam ab89) with Protein A or G agarose beads (Millipore 16-125, 16-201) in the presence or absence of 100 μg/ml ethidium bromide. Beads were washed 4 times in IP buffer and bound proteins eluted into SDS loading buffer (Laemmli) by heating at 90°C for 5 mins. Western blotting was performed using the following additional antibodies; anti-MRE11 (Abcam ab214), anti-NBS1 (Santa Cruz sc-11431).

### Terminal restriction fragment analysis

Telomere length was determined by terminal restriction length analysis [37]. 5μg of high molecular weight DNA was digested with HinfI and RsaI then separated by pulse field gel electrophoresis in 1% agarose and 0.5xTBE at 6V/cm for 15hrs with switch times of 0.1–20 secs for mouse and 0.1–6.0 secs for human DNA. After blotting the filter was probed with radioactively labelled telomeric sequence.

### Immunofluorescence and telomere FISH

Cells grown on coverslips were prepared for IF by standard procedures. The following antibodies were used for immunostaining: anti-ATRX (Santa Cruz sc-15408); anti-ATRX 39f [38]; anti MRE11 (Abcam ab214); anti-MRE11 (Calbiochem PC388); anti-RAD50 (Abcam ab89); anti-PCNA (Santa Cruz sc-9857); anti-RPA32 (S33) (Bethyl A300-246A); anti-NBS1 (BD Biosciences 611871); anti-53BP1 (Novus Biologicals NB100-305); For MRN co-localisation studies cells were pre-permeabilised prior to fixation with ice cold 0.5% Triton X-100. For MRE11/ATRX/PCNA co-localisation in mES cells, cells were attached to coverslips via poly-l-lysine treatment prior to pre-permeabilisation. Telomere FISH was performed subsequent to antibody incubation using Telomere PNA FISH Kit/Cy3 (Dako K5326).

### Bioinformatics analysis

Image analysis was done using custom perl processing scripts that called various ImageJ macros to allow filtering and automatic thresholding methods to segment nuclei and then identify foci per nucleus. The JACoP [39] plugin was used to examine coincident colocalisation and distance based colocalisation (DBC). Using ImageJ macros and Perl scripts a montage for each cell was generated which included the segmented nuclei, auto-thresholded images for each channel, original grey scale images for each channel and the JACoP DBC and CC. The images were visualised and analysed using the HTML5 PivotViewer (manuscript in preparation). The entire process was automated and all scripts are available on request. PivotViewer allowed the rapid analysis and review of the many hundreds of images generated from these analyses.

### G4 unwinding assay

This assay was performed according to a previously published method [40].

### Cell survival assay

150-300 mES cells were grown for 24h before treatment with the specified DNA damaging agents or replication inhibitors. After 3 or 4 days, colonies were stained with 1% w/v methylene blue 50% ethanol and counted.

### DNA fibre analysis

For fibre analysis mES cells were incubated with 25 μM IdU for 10 mins or 90 mins in the presence of 1 mM hydroxyurea, followed by a 10 or 20 min incubation with 250 μM CldU respectively. For assessment of fork processivity mES cells were incubated with IdU for 10 mins followed by a 40 min incubation with CldU in the presence of 1 mM hydroxyurea. Spreading and visualisation of fibres was performed as previously described [17] **.**

## Supporting Information

Figure S1 (A) Representative images of cell cycle profile for *Atrx^flox^* and *Atrx^null^* mES cells following 8 hours release from aphidicolin block. Black box shows late replicating population. (B) Percentage of late replicating cells as determined by FACs following 2, 6 and 8 hours post release from aphidicolin block. Error bars indicate ± SEM from three independent experiments (C) Immunoblot and quantitation (D) to assess levels of gamma-H2AX in histones purified from *Atrx^flox^* and *Atrx^null^* mES cells (clone 2) at the indicated time points. This showed an elevated DDR, reflected by elevated gamma-H2AX, in *Atrx^null^* cells as compared to the *Atrx^flox^* cells. U  =  unsynchronised cells. Histone H3 is shown as the loading control. Error bars indicate ± SEM. (E) Results of fibre analysis showing relative frequencies of replication intermediates in in *Atrx^flox^* and *Atrx^null^* mES cells (clone 2) with hydroxyurea treatment during the IdU pulse.
(TIF)

Figure S2 Representative images for immunofluorescence in wildtype mES cells showing 3 way co-localisation between PCNA, MRE11 and ATRX using an N-terminal specific ATRX antibody. Nuclei are outline with a dashed white line.
(TIF)

Figure S3 Unwinding assay for G4 DNA. Recombinant ATRX [41] was compared with BLM for its ability to unwind a G4 substrate. Both proteins were used in a 2 fold dilution series. ATRX binds G4 giving rise to a shifted signal but unlike BLM does not unwind the G4 DNA.
(TIF)
