# Supplementary figures and images for: ATRX Dysfunction Induces Replication Defects in Primary Mouse Cells
Source: PLoS One. 2014 Mar 20;9(3):e92915. doi: 10.1371/journal.pone.0092915 (PMC3961441; doi:10.1371/journal.pone.0092915)

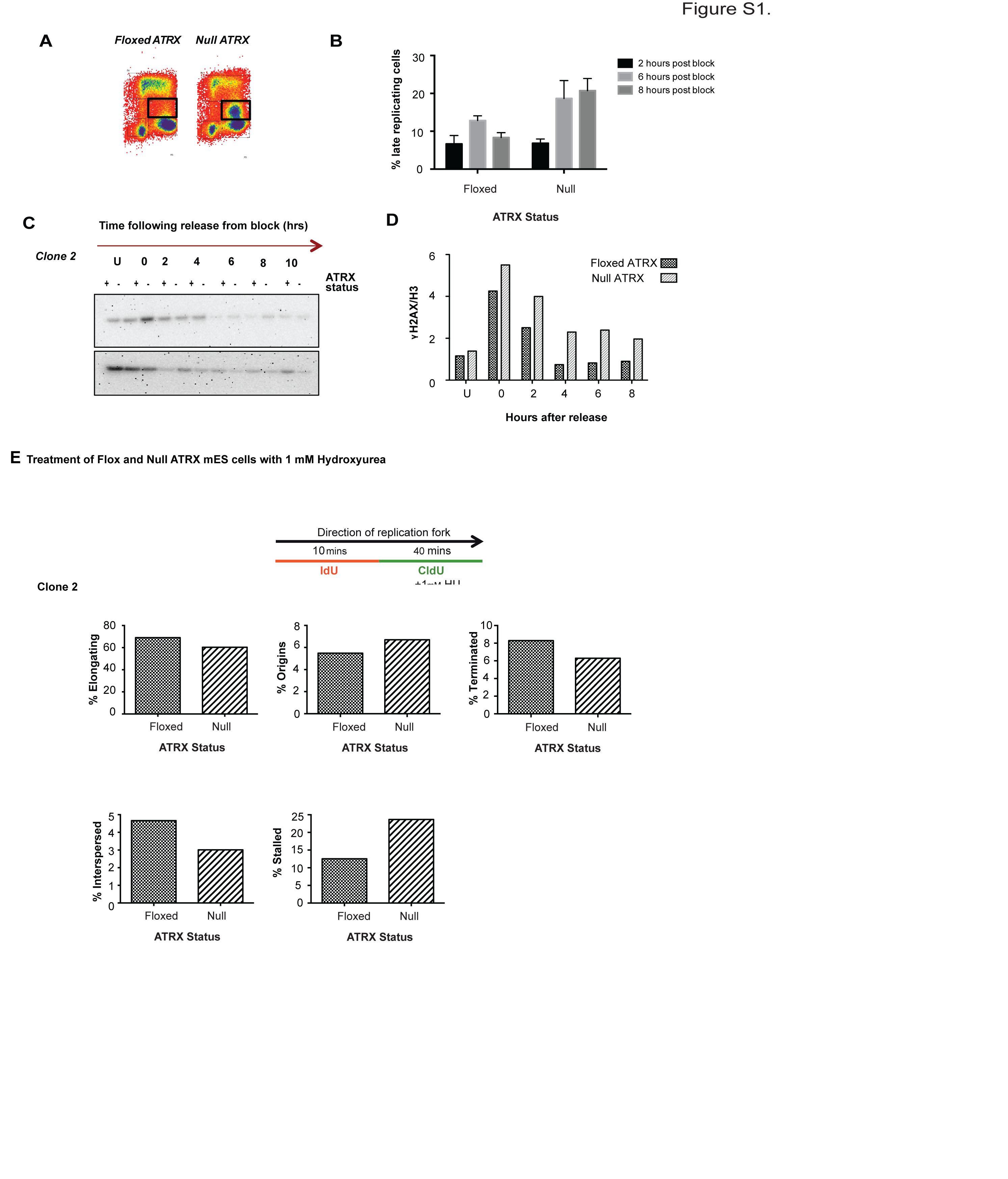

Supplement: Figure S1 — (A) Representative images of cell cycle profile for Atrxflox and Atrxnull mES cells following 8 hours release from aphidicolin block. Black box shows late replicating population. (B) Percentage of late replicating cells as determined by FACs following 2, 6 and 8 hours post release from aphidicolin block. Error bars indicate ± SEM from three independent experiments (C) Immunoblot and quantitation (D) to assess levels of gamma-H2AX in histones purified from Atrxflox and Atrxnull mES cells (clone 2) at the indicated time points. This showed an elevated DDR, reflected by elevated gamma-H2AX, in Atrxnull cells as compared to the Atrxflox cells. U = unsynchronised cells. Histone H3 is shown as the loading control. Error bars indicate ± SEM. (E) Results of fibre analysis showing relative frequencies of replication intermediates in in Atrxflox and Atrxnull mES cells (clone 2) with hydroxyurea treatment during the IdU pulse. (TIF) [file pone.0092915.s001.tif]

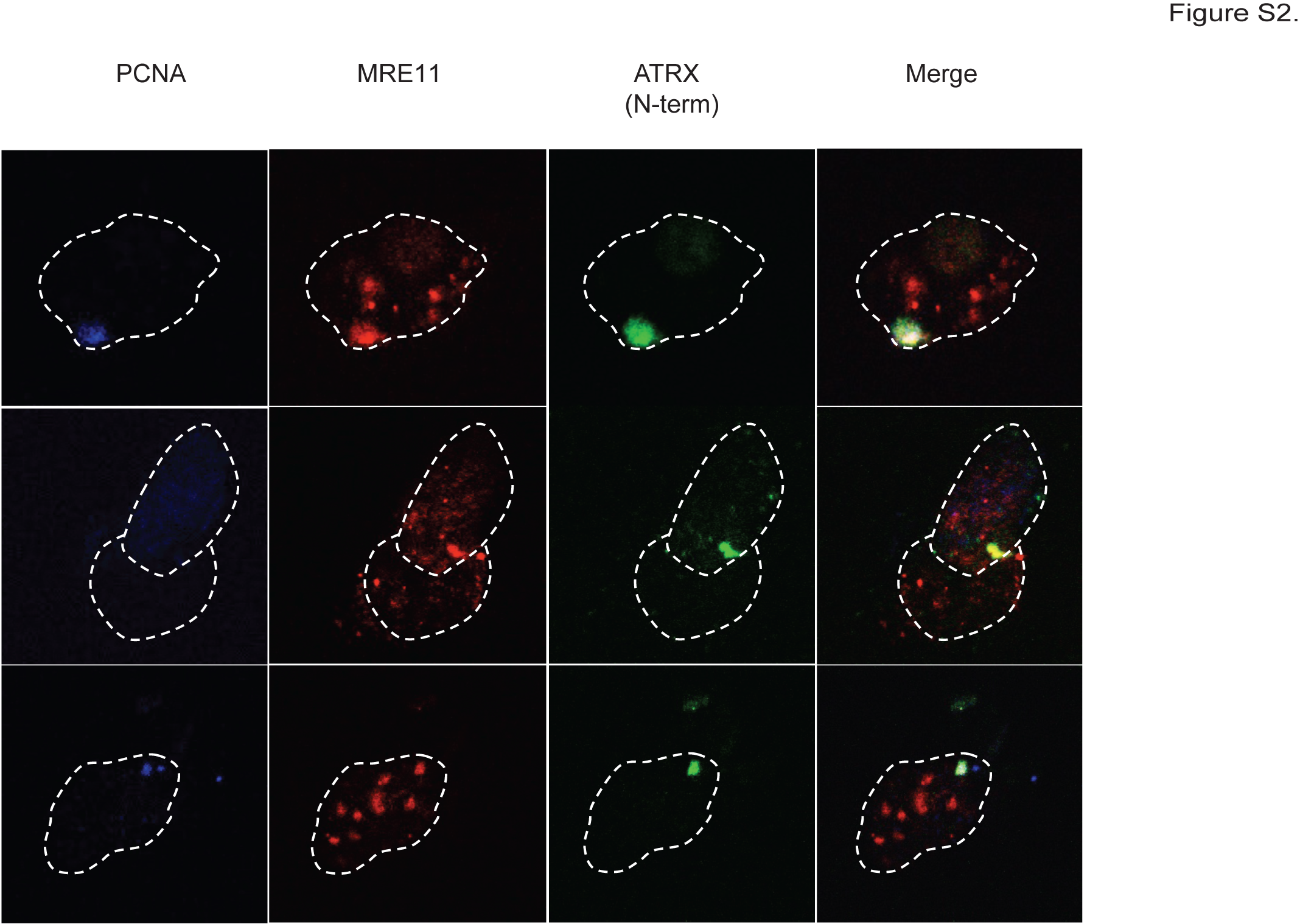

Supplement: Figure S2 — Representative images for immunofluorescence in wildtype mES cells showing 3 way co-localisation between PCNA, MRE11 and ATRX using an N-terminal specific ATRX antibody. Nuclei are outline with a dashed white line. (TIF) [file pone.0092915.s002.tif]

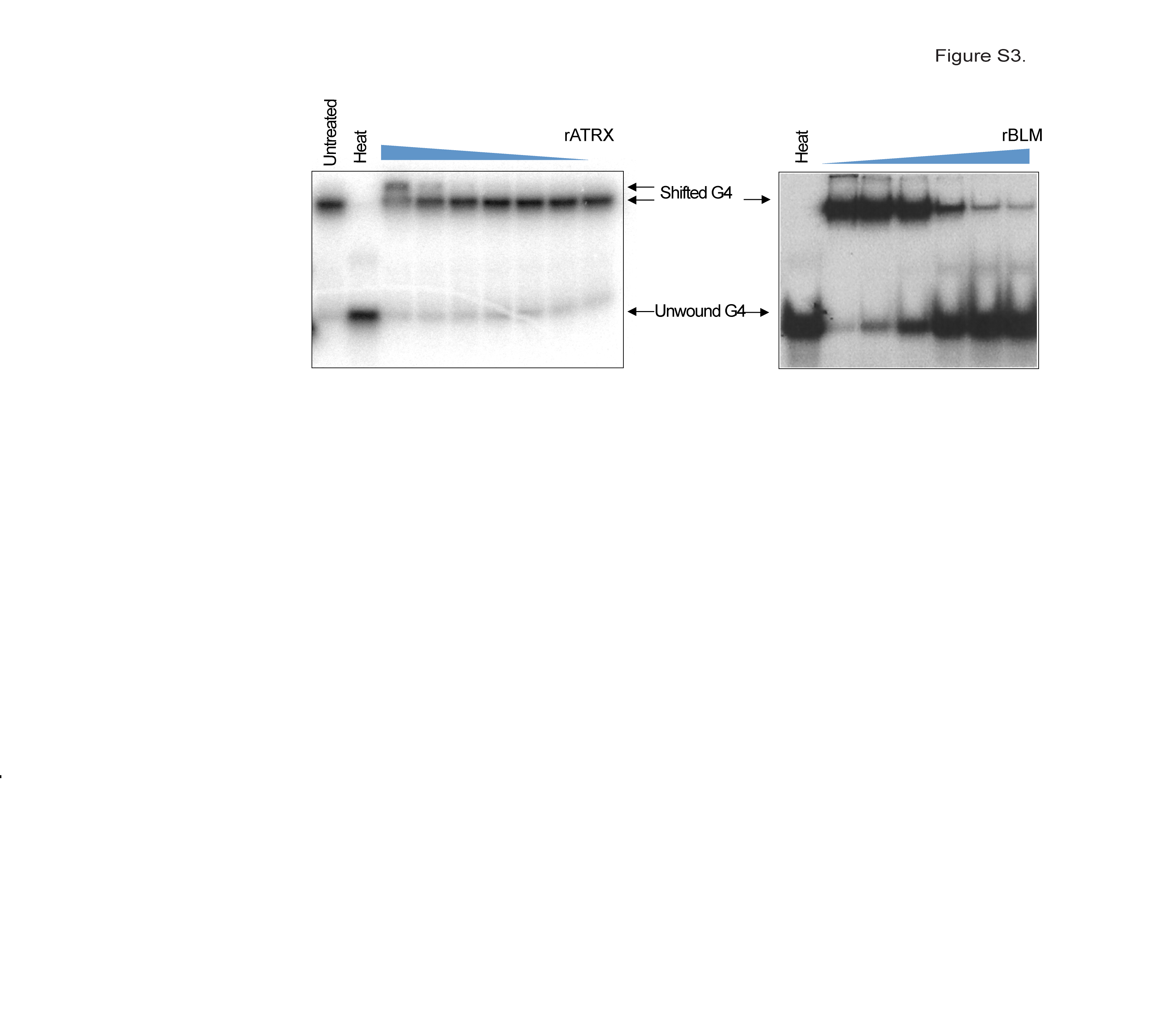

Supplement: Figure S3 — Unwinding assay for G4 DNA. Recombinant ATRX [41] was compared with BLM for its ability to unwind a G4 substrate. Both proteins were used in a 2 fold dilution series. ATRX binds G4 giving rise to a shifted signal but unlike BLM does not unwind the G4 DNA. (TIF) [file pone.0092915.s003.tif]
